# Overweight, obesity and the risk of LADA: results from a Swedish case–control study and the Norwegian HUNT Study

**DOI:** 10.1007/s00125-018-4596-0

**Published:** 2018-03-27

**Authors:** Rebecka Hjort, Emma Ahlqvist, Per-Ola Carlsson, Valdemar Grill, Leif Groop, Mats Martinell, Bahareh Rasouli, Anders Rosengren, Tiinamaija Tuomi, Bjørn Olav Åsvold, Sofia Carlsson

**Affiliations:** 1 Unit of Epidemiology, Institute of Environmental Medicine, Karolinska Institutet, Box 210, 171 77 Stockholm, Sweden; 2 Department of Clinical Sciences in Malmö, Clinical Research Centre, Lund University, Malmö, Sweden; 3 Department of Medical Sciences, Uppsala University, Uppsala, Sweden; 4 Department of Clinical and Molecular Medicine, NTNU, Norwegian University of Science and Technology, Trondheim, Norway; 5 Department of Endocrinology, St Olavs Hospital, Trondheim University Hospital, Trondheim, Norway; 6 Finnish Institute of Molecular Medicine, University of Helsinki, Helsinki, Finland; 7 Department of Public Health and Caring Sciences, Uppsala University, Uppsala, Sweden; 8 Division of Endocrinology, Abdominal Center, Helsinki University Hospital, Research Program for Diabetes and Obesity, University of Helsinki, Helsinki, Finland; 9 Folkhälsan Research Center, Helsinki, Finland; 10 K.G. Jebsen Center for Genetic Epidemiology, Department of Public Health and Nursing, NTNU, Norwegian University of Science and Technology, Trondheim, Norway

**Keywords:** ANDIS, ANDiU, Body mass index, Case–control study, ESTRID, HUNT Study, LADA, Latent autoimmune diabetes in adults, Prospective study, Type 2 diabetes

## Abstract

**Aims/hypothesis:**

Excessive weight is a risk factor for type 2 diabetes, but its role in the promotion of autoimmune diabetes is not clear. We investigated the risk of latent autoimmune diabetes in adults (LADA) in relation to overweight/obesity in two large population-based studies.

**Methods:**

Analyses were based on incident cases of LADA (*n* = 425) and type 2 diabetes (*n* = 1420), and 1704 randomly selected control participants from a Swedish case–control study and prospective data from the Norwegian HUNT Study including 147 people with LADA and 1,012,957 person-years of follow-up (1984–2008). We present adjusted ORs and HRs with 95% CI.

**Results:**

In the Swedish data, obesity was associated with an increased risk of LADA (OR 2.93, 95% CI 2.17, 3.97), which was even stronger for type 2 diabetes (OR 18.88, 95% CI 14.29, 24.94). The association was stronger in LADA with low GAD antibody (GADA; <median) (OR 4.25; 95% CI 2.76, 6.52) but present also in LADA with high GADA (OR 2.14; 95% CI 1.42, 3.24). In the Swedish data, obese vs normal weight LADA patients had lower GADA levels, better beta cell function, and were more likely to have low-risk HLA-genotypes. The combination of overweight and family history of diabetes (FHD) conferred an OR of 4.57 (95% CI 3.27, 6.39) for LADA and 24.51 (95% CI 17.82, 33.71) for type 2 diabetes. Prospective data from HUNT indicated even stronger associations; HR for LADA was 6.07 (95% CI 3.76, 9.78) for obesity and 7.45 (95% CI 4.02, 13.82) for overweight and FHD.

**Conclusions/interpretation:**

Overweight/obesity is associated with increased risk of LADA, particularly when in combination with FHD. These findings support the hypothesis that, even in the presence of autoimmunity, factors linked to insulin resistance, such as excessive weight, could promote onset of diabetes.

**Electronic supplementary material:**

The online version of this article (10.1007/s00125-018-4596-0) contains peer-reviewed but unedited supplementary material, which is available to authorised users.

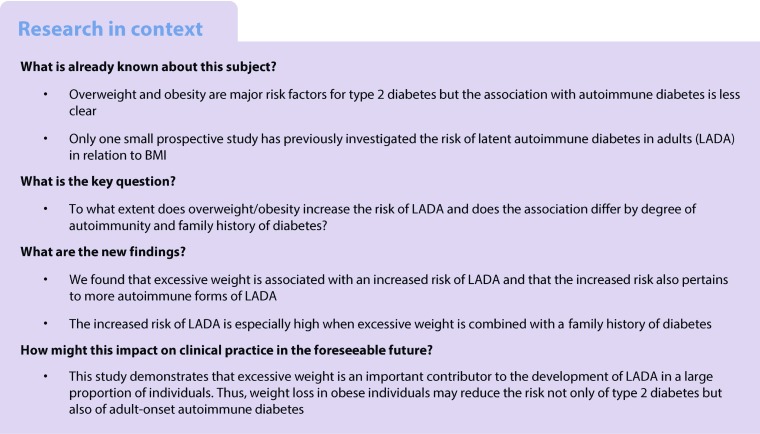



## Introduction

Overweight and obesity are major risk factors for type 2 diabetes [1], and the association between excessive weight and insulin resistance is well known. Several mediating pathways have been proposed, including ectopic lipid accumulation and lipotoxicity, and the release of proinflammatory cytokines from visceral fat tissue [2].

Type 1 diabetes has been viewed as a non-obese form of diabetes, but this was challenged by the accelerator hypothesis [3], which proposes that obesity accelerates disease onset and, further, that insulin resistance is a common underlying feature of all types of diabetes [3]. Insulin resistance has also been postulated to be an independent risk factor for type 1 diabetes [4, 5]. Adiposity could potentially affect risk via beta cell autoimmunity; adipokines, which are secreted from excessive fat tissue, have been shown to be involved in various immune-mediating processes [6]. Subsequent prospective studies have reported a twofold increased risk of type 1 diabetes in obese children [7] and obese adults [8], while others find no association [9]. An association is further supported by the coincident increases in childhood obesity and type 1 diabetes incidence [10, 11].

Latent autoimmune diabetes in adults (LADA) is an autoimmune form of diabetes with features of type 2 diabetes, including adult onset and insulin resistance [12]. Autoimmunity is typically less pronounced than in type 1 diabetes, which implies that insulin resistance, increasing the beta cell demand, may play a key role in the promotion of LADA. In line with this, data from cross-sectional studies [13–19] suggest that individuals with LADA tend to have higher BMI than those with type 1 diabetes, but lower than those with type 2 diabetes. Interestingly, the clinical phenotype of LADA is known to vary by degree of autoimmunity, with less autoimmune individuals being more type 2-like. Hence, the role of overweight in the development of LADA may depend on the severity of the underlying autoimmune process. Only one prospective study based on 11 years follow-up of the Norwegian Nord-Trøndelag Health Study (HUNT Study) estimated the risk of LADA in relation to overweight/obesity [20]. This study was based on only 81 individuals; hence, the influence of excessive weight on more or less autoimmune forms of LADA could not be explored, and confounding control was limited. Other aspects that remain to be addressed include interaction between overweight and family history of diabetes (FHD), which is a strong indicator of type 2 diabetes risk [21], whether the shape of association is linear or not, and the preventive potential of overweight in the aetiology of LADA.

Our aim was to describe the association between overweight and obesity and LADA compared with type 2 diabetes, taking into account degree of autoimmunity and potential interaction with FHD. We used updated prospective data from the HUNT Study, including 22 years of follow-up, and newly collected data from a Swedish case–control study with incident cases.

## Methods

### The ESTRID study

#### Study population and design

The Epidemiological Study of Risk Factors for LADA and Type 2 Diabetes (ESTRID) is an ongoing population-based case–control study [22]. In short, ESTRID is a substudy of All New Diabetics In Scania (ANDIS; http://andis.ludc.med.lu.se), an extensive diabetes study aimed at characterising clinical and genetic factors in incident cases in Scania. Since 2010, we have recruited all incident cases of LADA and a random sample of those with type 2 diabetes (four for every one person with LADA) from ANDIS. Since 2012, we have also included individuals from ANDiU (All New Diabetics in Uppsala County; www.andiu.se/), a sister study to ANDIS. Control participants (six for every one person with LADA [≥35 years of age]) without diabetes are randomly selected from the national population register and matched to the case for county and time of recruitment (incident density sampling) [23].

The analytical sample for the present study consisted of all individuals recruited until July 2016 and with complete information on BMI, age, sex, FHD, physical activity and smoking (98.2% of the study sample): 425 individuals with LADA, 1420 individuals with type 2 diabetes and 1704 control participants (95% of participants came from Scania and 5% came from Uppsala). The response rate was 80% for the individuals with LADA and type 2 diabetes and 64% for control participants. ESTRID was approved by the ethical review board in Stockholm and all participants gave written informed consent.

#### Case definition and biochemical analyses

At diagnosis, blood samples were collected from all individuals and analysed for GAD antibody (GADA) in serum using ELISA (RSR, Cardiff, UK). At the cut-off level for positivity (10 U/ml), sensitivity was 84% and specificity 98% [24]. Fasting C-peptide was assessed in plasma using IMMULITE 2000 (Siemens Healthcare Diagnostics, Llanberis, UK) or Cobas e 601 (Roche Diagnostics, Mannheim, Germany).

Individuals with LADA had diagnosis ≥35 years of age, were GADA positive (≥10 U/ml) and had C-peptide levels above the lower limit for the normal range ≥0.2 nmol/l (IMMULITE) or ≥0.3 nmol/l (Cobas e 601). Type 2 diabetes was defined as onset ≥35 years of age, GADA negativity (<10 U/ml) and C-peptide levels >0.6 nmol/l (IMMULITE) or ≥0.72 nmol/l (Cobas e 601). DNA was analysed using iPLEX Gold technology (Sequenom Laboratories, San Diego, CA, USA). Three SNPs in the MHC region (rs3104413, rs2854275, rs9273363) were combined to identify high- and low-risk HLA-DR and HLA-DQ genotypes associated with autoimmunity [25], according to previously used methods [26]. Missing genotypes were completed using imputed data from an ANDIS subset genotyped on Infinium CoreExome v1.1 (Illumina, San Diego, CA, USA), imputed based on the Haplotype Reference Consortium (http://www.haplotype-reference-consortium.org/; version r1.1 2016) reference panel. HOMA was used to estimate insulin resistance, insulin sensitivity and beta cell function based on the relationship between fasting values of C-peptide and plasma glucose [27]. No genetic or clinical information was available for the control participants.

#### BMI and covariates

Case and control participants answered an extensive questionnaire at inclusion. For those with LADA or type 2 diabetes, this was done as close to diagnosis as possible (median 5 months), with careful instructions to report lifestyle as it was prior to diagnosis. Current BMI was based on self-reported weight and height, which shows high correlation (*r* = 0.92) with BMI based on measurements taken at diagnosis (those with LADA or type 2 diabetes). BMI was categorised as: normal weight <25 kg/m^2^, overweight 25–29.9 kg/m^2^ and obese ≥30 kg/m^2^ (WHO). BMI at age 20 years was calculated based on self-reported information on weight at age 20 years (80.4% of the study sample could recall this information) and current height. FHD was obtained from questions on diabetes in first-degree relatives (mother, father, sisters, brothers and children). Relatives with onset <40 years of age and with insulin treatment were considered to have type 1 diabetes, otherwise they were judged to have type 2 diabetes. Physical activity level (sedentary, low, moderate or high activity) was assessed from validated questions [28] on leisure time activity. Individuals were categorised based on highest achieved education (primary school, upper secondary school, university) and through detailed questions on lifetime smoking as never, former or current smokers. Alcohol habits were categorised into four groups (ranging from abstainers to high consumers), based on questions on amount and frequency of wine, beer and liquor intake.

### The HUNT Study

#### Study population and design

In the county of Nord-Trøndelag, all residents ≥20 years of age were invited to participate in the HUNT Study on three occasions between 1984 and 2008: HUNT1 (1984–1986), HUNT2 (1995–1997) and HUNT3 (2006–2008) [29]. At each survey, data for participants were gathered from clinical examinations, anthropometrical measurements and comprehensive questionnaires with questions on general health, FHD and lifestyle. Analyses were based on all individuals who participated in at least two surveys, were free of diabetes at baseline and with complete information on BMI, age, sex, FHD, physical activity and smoking (*n* = 56,549). The HUNT Study was approved by the Norwegian Data Protection Authority and the Regional Committee for Medical and Health Research Ethics and participants gave informed consent.

#### Case definition

Incident cases were identified by self-report of diabetes and age at diagnosis. This self-report has high validity when compared with medical records [30]. Individuals with self-reported diabetes at HUNT2 or HUNT3 were invited for fasting blood sampling. Level of GADA, reported as an index value in relation to standard serum, was measured in fasting serum samples by immunoprecipitation radioligand assay using translation-labelled [^3^H]GAD65 as a labelled reagent (Novo Nordisk, Bagsværd, Denmark). The sensitivity and specificity of the assay were 0.64 and 1.00 at cut-off ≥0.08 [31]. Individuals were classified as having LADA if they were aged ≥35 years at diagnosis and GADA positive (≥0.08 antibody index [WHO; ≥43 U/ml]; *n* = 147). This implies that we have included individuals with adult-onset type 1 diabetes. The proportion accounted for by these individuals is likely to be small as, when we used information on treatment (available for 83.5% of the total study population), 82.7% (*n* = 105) of those with GADA positivity did not report insulin treatment during the year of diagnosis. For convenience, this group will be referred to as LADA; subanalysis based on a stricter definition of LADA (no insulin treatment) has been conducted. Individuals with type 2 diabetes were ≥35 years of age and GADA negative (<43 U/ml; *n* = 2002). C-peptide (nmol/l) (not from time of diagnosis) was measured in fasting serum samples and analysed by RIA (Diagnostic Systems Laboratories, Webster, TX, USA). HOMA indicators were calculated based on fasting C-peptide and glucose as described above.

#### BMI, WHR and covariates

BMI was calculated from weight and height measured at the clinical examination. Waist and hip circumference (only available from baseline in HUNT2) and height were used to calculate WHR and waist-to-height ratio (WHtR). The measures were dichotomised according to previously used risk levels [32]. Those with self-reported FHD in any of the three surveys were considered to have FHD. Baseline information (HUNT1 or HUNT2) was used to classify individuals according to leisure-time physical activity (sedentary, low, moderate or high activity), highest-attained education (primary school, upper secondary school, university), smoking status (never, former, current) and alcohol consumption (abstainers, low, moderate or high consumers).

### Statistical analyses

Baseline characteristics were expressed as proportions, means (SD), or medians (interquartile range [IQR]). Two-sided *p* values were calculated using *χ*^2^ (proportions), Student’s *t* (means) and Kruskal–Wallis (medians). ORs with 95% CIs were calculated by conditional logistic regression for case–control data (ESTRID) and HRs with CIs were calculated by proportional Cox regression for prospective data (HUNT). As control participants were sampled with an incidence density method, the ORs can be interpreted as incidence rate ratios [23]. In HUNT, person-years were calculated from age at study entry until age at onset of diabetes, death or age at end of the follow-up (either HUNT2 in 1997 or HUNT3 in 2008), whichever came first. Time-dependent variables were used, hence for individuals participating both in HUNT1 and HUNT2, information on exposure and covariates was updated at the second time of participation.

To explore the relationship between BMI and diabetes, we used restricted cubic spline models to allow fitting of a smooth curve without assumption about linearity [33], modelled with five knots at equally spaced percentiles of the marginal distribution of BMI. BMI was truncated below 15 kg/m^2^ and above 45 kg/m^2^ to remove the influence of outliers. The relationship between BMI and insulin resistance (log_*e*_ HOMA-IR) and log_*e*_ GADA was assessed by linear regression. Interaction was defined as departure from additivity of effects [34] and tested by calculating attributable proportion due to interaction together with 95% CI [35]. Population-attributable risk (PAR) was calculated with the formula: *p*(1-[1/RR]) where *p* is the prevalence (%) of the risk factor of interest among cases and RR is the adjusted OR (ESTRID) or HR (HUNT) [36]. All analyses were adjusted for by age (underlying timescale in the Cox model), sex, first-degree FHD, physical activity and smoking. Adjustment for alcohol intake and education had minor effects on the risk estimates (<10% change) and were not included in the final model. Individuals with LADA were stratified by median GADA level (196.0 U/ml [ESTRID] and 134.4 U/ml [HUNT]), referred to in the paper as LADA^low^ and LADA^high^. Statistical Analysis Software (SAS) 9.4 (SAS Institute, Cary, NC, USA) or Stata Statistical Software 14 (StataCorp, College Station, TX, USA) (for calculating splines) were used for the statistical analyses.

## Results

### Characteristics

In both populations, individuals with LADA were younger at diagnosis, had lower C-peptide concentrations and were more often on insulin treatment than individuals with type 2 diabetes (Table 1). In ESTRID, individuals with LADA had a lower level of insulin resistance (HOMA) and had a higher proportion of high-risk HLA genotypes and FHD of type 1 diabetes. Individuals with LADA were leaner than those with type 2 diabetes, whereas in HUNT, there was no corresponding difference (Table 1). However, mean WHR was higher in individuals with type 2 diabetes. Comparing LADA^low^ and LADA^high^, the former group displayed higher concentrations of C-peptide and better beta cell function but a higher level of insulin resistance (ESM Table 1).

**Table 1:** Characteristics of study participants

Characteristics	HUNT				ESTRID			
	No diabetes	Type 2 diabetes	LADA	*p* ^a^	Control participants	Type 2 diabetes	LADA	*p* ^a^
Number of individuals	54,440	2002	147		1704	1420	425	
Women, %	53.2	47.3	51.7	0.3028	51.9	39.2	45.7	0.0181
Age at diagnosis, years (at inclusion for control participants), mean (SD)^b^	–	60.9 (10.9)	59.9 (11.1)	0.3039	58.4 (13.5)	63.2 (10.3)	59.0 (12.3)	<0.0001
Age, years, at baseline (HUNT), mean (SD)	48.3 (15.8)	54.8 (11.0)	54.4 (11.2)	0.6928	–	–	–	–
BMI, kg/m^2^, mean (SD)	25.5 (3.8)	29.8 (4.5)	29.2 (4.9)	0.1704	25.9 (4.2)	31.2 (5.4)	28.1 (5.3)	<0.0001
WHR, mean (SD)^c^	0.84 (0.08)	0.90 (0.07)	0.87 (0.07)	0.0260	–	–	–	–
Any first-degree FHD, %	24.2	57.1	48.3	0.0379	24.4	49.8	45.2	0.0951
FHD-T2D, %	–	–	–	–	22.65	47.61	36.71	<0.0001
FHD-T1D, %	–	–	–	–	2.58	5.00	11.29	<0.0001
With insulin treatment, %	–	3.4	17.3	<0.0001	–	5.9	41.2	<0.0001
C-peptide, nmol/l, median (IQR)^d^	–	0.86 (0.60)	0.57 (0.78)	<0.0001	–	1.20 (0.65)	0.69 (0.67)	<0.0001
GADA, U/ml, median (IQR)	–	–	134.4 (521.4)	–	–	–	196.0 (224.0)	–
HOMA-IR, median (IQR)^d^	–	2.20 (1.60)	2.10 (1.70)	0.1119	–	3.50 (2.20)	2.70 (2.60)	<0.0001
HOMA-β, median (IQR)^d^	–	64.5 (49.2)	59.0 (50.8)	0.4109	–	68.1 (49.8)	37.8 (53.6)	<0.0001
HOMA-S, median (IQR)^d^	–	45.2 (30.7)	47.2 (49.0)	0.1263	–	28.2 (16.4)	36.9 (32.8)	<0.0001
High-risk HLA, %^e^	–	–	–	–	–	31.1	61.4	<0.0001
Low-risk HLA, %^e^	–	–	–	–	–	45.0	21.9	<0.0001

^a^*p* for difference between LADA and type 2 diabetes

^b^Median 5 months after diabetes diagnosis for cases in ESTRID

^c^Information only available from baseline at HUNT2 (1995–1997)

^d^Clinical information was available for 92.6% of the individuals in ESTRID (LADA *n* = 394, type 2 diabetes *n* = 1315) and 70.7% of participants in HUNT (LADA *n* = 118, type 2 diabetes *n* = 1401)

^e^Genetic information was available for 90.4% of the individuals in ESTRID (LADA *n* = 389, type 2 diabetes *n* = 1278)

T1D, type 1 diabetes; T2D, type 2 diabetes

### Overweight, obesity and LADA

In ESTRID, the OR for LADA was 2.93 (95% CI 2.17, 3.97) among obese compared with normal weight participants (Table 2). The association seemed stronger in LADA^low^ (OR 4.25, 95% CI 2.76, 6.52) than in LADA^high^ (OR 2.14, 95% CI 1.42, 3.24). Prospective data from HUNT indicated similar but stronger associations; the HR associated with obesity was 6.07 (95% CI 3.76, 9.78) for LADA, 10.00 (95% CI 4.34, 23.03) for LADA^low^ and 4.58 (95% CI 2.49, 8.45) for LADA^high^. Abdominal obesity (HUNT) increased the risk of LADA nearly twofold (HR 1.89, 95% CI 1.03, 3.46) measured with WHR and threefold (HR 3.14, 95% CI 1.56, 6.30) measured with WHtR (Table 2) (HUNT). Results from HUNT were similar with a stricter definition of LADA (no insulin treatment); HR was 6.63 (95% CI 3.67, 12.00) for obesity. LADA was also associated with weight change over time; for every unit increase in BMI since age 20 years, OR increased by 10% (OR 1.10, 95% CI 1.07, 1.14) (Table 2). The association between BMI and LADA was similar in men and women (ESM Tables 2 and 3).

**Table 2:** Overweight/obesity and LADA. Results from ESTRID 2010–2016 and HUNT 1984–2008

Variable	No. control participants/person-years	LADA		LADA^high^		LADA^low^		Type 2 diabetes	
		No. cases	OR/HR^a^ (95% CI)	No. cases	OR/HR^a^ (95% CI)	No. cases	OR/HR^a^ (95% CI)	No. cases	OR/HR^a^ (95% CI)
ESTRID									
BMI (kg/m^2^)									
<25	777	125	1	68	1	44	1	101	1
25–29.9	671	165	1.38 (1.06, 1.80)	77	1.26 (0.88, 1.79)	78	1.66 (1.11, 2.48)	590	5.14 (3.99, 6.61)
≥30	256	135	2.93 (2.17, 3.97)	52	2.14 (1.42, 3.24)	75	4.25 (2.76, 6.52)	729	18.88 (14.29, 24.94)
Per 1 kg/m^2^ increase	1704	425	1.10 (1.07, 1.13)	197	1.07 (1.04, 1.11)	197	1.13 (1.09, 1.17)	1420	1.29 (1.26, 1.32)
Per 1 kg/m^2^ increase since age 20	1412	339	1.10 (1.07, 1.14)	155	1.03 (0.99, 1.08)	159	1.16 (1.11, 1.20)	1103	1.27 (1.23, 1.30)
HUNT									
BMI (kg/m^2^)									
<25	494,231	26	1	19	1	7	1	238	1
25–29.9	401,641	64	2.16 (1.36, 3.43)	29	1.51 (0.84, 2.74)	35	3.81 (1.68, 8.66)	915	3.30 (2.86, 3.81)
≥30	117,085	57	6.07 (3.76, 9.78)	26	4.58 (2.49, 8.45)	31	10.00 (4.34, 23.03)	849	9.83 (8.49, 11.38)
Per 1 kg/m^2^ increase	1,012,957	147	1.16 (1.13, 1.20)	74	1.15 (1.10, 1.20)	73	1.18 (1.13, 1.23)	2002	1.19 (1.18, 1.20)
Per 1 kg/m^2^ increase over time^b^	359,732	48	1.18 (1.03, 1.34)	33	1.07 (0.91, 1.26)	15	1.30 (1.07, 1.58)	943	1.22 (1.18, 1.25)
WHR^c^									
<0.85 (W), <0.90 (M)	329,347	27	1	20	1	7	1	388	1
≥0.85 (W), ≥0.90 (M)	134,323	24	1.89 (1.03, 3.46)	15	1.70 (0.81, 3.56)	9	2.36 (0.80, 6.98)	650	3.57 (3.11, 4.09)
WHtR^c^									
<0.50	229,054	11	1	9	1	2	1	134	1
≥0.50	234,035	40	3.14 (1.56, 6.30)	26	2.63 (1.18, 5.83)	14	5.45 (1.19, 25.04)	903	5.08 (4.21, 6.12)

^a^ORs (ESTRID) and HRs (HUNT) adjusted for age, sex, FHD, smoking and physical activity

^b^Change in BMI from HUNT1 (1984–1986) until baseline in HUNT2 (1995–1997)

^c^Information only available from baseline in HUNT2 (1995–1997)

M, men; No., number; W, women

### Overweight, obesity and type 2 diabetes

The association between overweight/obesity and type 2 diabetes was stronger than for LADA, obesity was associated with OR of 18.88 (95% CI 14.29, 24.94) in ESTRID and HR was 9.83 (95% CI 8.49, 11.38) in HUNT (Table 2). Abdominal obesity was associated with type 2 diabetes, HR 3.57 (95% CI 3.11, 4.09; WHR) and HR 5.08 (95% CI 4.21, 6.12; WHtR). For every BMI unit increase since age 20 years, OR for type 2 diabetes increased by 27% (OR 1.27, 95% CI 1.23, 1.30) (ESTRID).

### Restricted cubic spline analyses

**Fig. 1:**
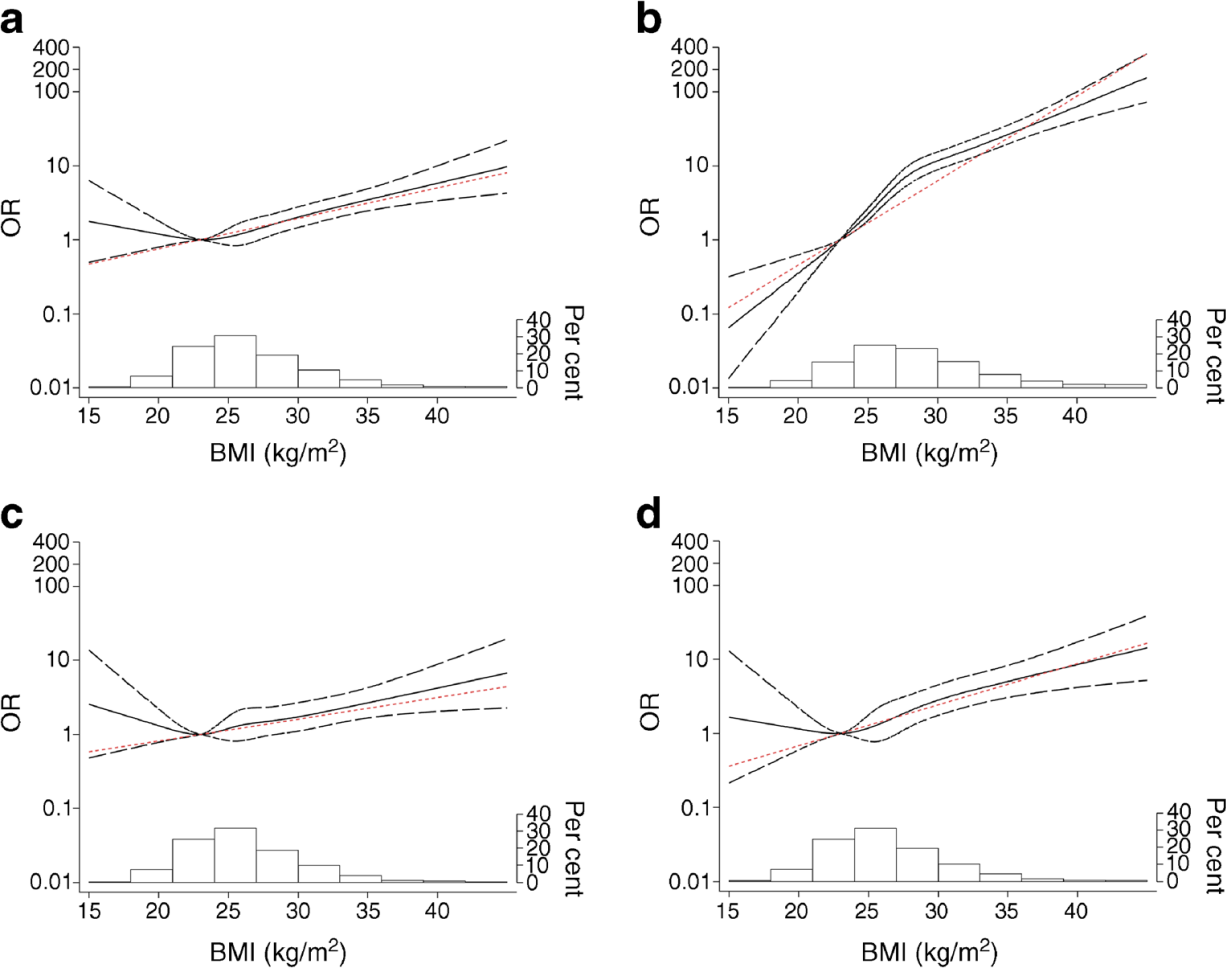
ORs with 95% CIs for (**a**) LADA, (**b**) type 2 diabetes, (**c**) LADA^high^, and (**d**) LADA^low^ by BMI (kg/m^2^) fitted with restricted cubic splines using data from ESTRID 2010–2016. The reference value is BMI 23 kg/m^2^ and models were adjusted for age, sex, FHD, physical activity level and smoking. Black solid lines represent the spline line, long dashed lines represent the 95% CIs of the spline line and the red dotted lines represent the linear line. The histogram at the bottom of each figure part represents the distribution of BMI in the study population. The left *y*-axes are on a log_*e*_ scale

Restricted cubic spline models were used to explore the potential linear relationship between BMI and diabetes (Fig. 1). For type 2 diabetes, a strong linear association was seen over the whole range of BMI with a slight levelling off above BMI 27 kg/m^2^. For LADA, a linear pattern was less pronounced with a tendency of a U-shaped relationship; however, above BMI 24 kg/m^2^ the OR increased exponentially. A similar shape was seen for both LADA^high^ and LADA^low^, but with an apparently steeper line for the latter group.

### Interaction between overweight and FHD

**Fig. 2:**
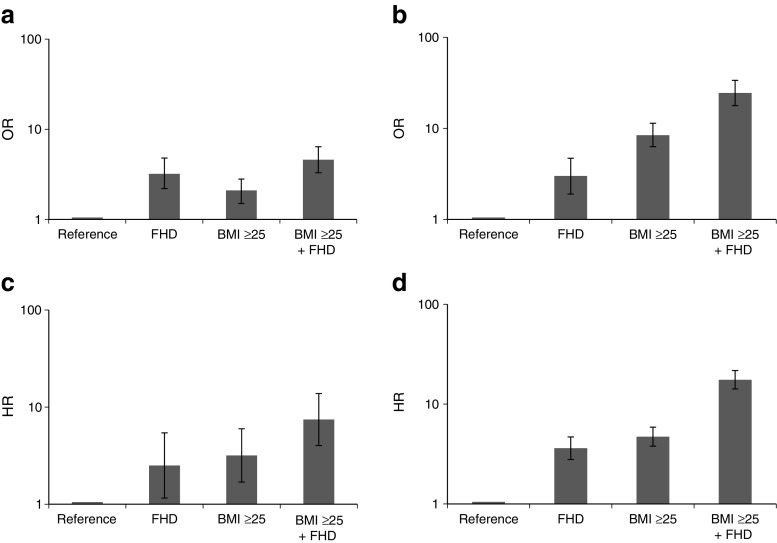
ORs for (**a**) LADA and (**b**) type 2 diabetes in ESTRID. HRs for (**c**) LADA and (**d**) type 2 diabetes in HUNT by combinations of overweight/obesity (BMI ≥ 25 kg/m^2^) and FHD. The reference is normal weight (BMI < 25 kg/m^2^) and no FHD. The *y*-axis is on the log_10_ scale and error bars are 95% CI

Individuals with a combination of FHD and overweight had OR 4.57 (95% CI 3.27, 6.39) for LADA and 24.51 (95% CI 17.82, 33.71) for type 2 diabetes (ESTRID). Corresponding HR estimates in HUNT were 7.45 (95% CI 4.02, 13.82) and 17.52 (95% CI 14.17, 21.66), respectively (Fig. 2). Interaction between FHD and overweight was seen for type 2 diabetes (attributable proportion 0.57, 95% CI 0.49, 0.66), but not for LADA (attributable proportion 0.06 95% CI −0.25, 0.37) in ESTRID. Results in HUNT were similar for type 2 diabetes (attributable proportion 0.58, 95% CI 0.53, 0.63), but stronger for LADA (attributable proportion 0.37, 95% CI 0.10, 0.64).

### Population-attributable risk

Calculations of PAR indicated that 31.0% (95% CI 20.2%, 39.5%) of all individuals with LADA and 81.8% (95% CI 78.7%, 84.1%) of all individuals with type 2 diabetes in the ESTRID study can be ascribed to overweight/obesity. Corresponding proportions in HUNT were 56.4% (95% CI 42.3%, 65.5%) (LADA) and 69.9% (95% CI 67.2%, 72.2%) (type 2 diabetes).

### Characteristics of individuals with LADA by category of BMI

In both populations, obese vs normal weight individuals with LADA had greater insulin production (C-peptide) and were less often receiving insulin treatment (ESM Tables 4 and 5). In ESTRID, obese individuals also had lower GADA levels, better beta cell function (HOMA) and a higher level of insulin resistance (HOMA). Similar tendencies were seen in HUNT. However, the differences were not significant (ESM Tables 4 and 5). In ESTRID, obese individuals with LADA were also more likely to have low-risk HLA genotypes and tended to less commonly have first-degree relatives with type 1 diabetes. BMI was positively associated with HOMA-IR (2.2% increase, *p* = 0.0002) and inversely associated with GADA (5.1% decrease*, p* < 0.0001) per BMI unit. In HUNT, results were similar for HOMA-IR (3.8% increase, *p* = 0.0077) but weaker for GADA (0.8% decrease, *p* = 0.6773).

## Discussion

Our findings using data from two large population-based studies indicate that overweight and obesity are associated with an increased risk of LADA and that the risk is highest in individuals with a combination of overweight and FHD. The association with obesity seemed strongest in LADA with low GADA, but was apparent also in LADA with higher GADA levels. The results indicate that LADA in 31–56% of individuals could be attributed to overweight/obesity, compared with 70–82% of all those with type 2 diabetes.

These findings fit with those of previous cross-sectional studies, which indicated that individuals with LADA tend to be obese but leaner than those with type 2 diabetes [13–19] and with previous reports of LADA being characterised by insulin resistance, but to a lesser extent than type 2 diabetes [12]. One previous study found that a majority of individuals with LADA have a lean phenotype [37]. One explanation of this somewhat conflicting result may be the use of a different age criterion (>25 years), as younger age at onset tends to be associated with a more type-1-like phenotype [38]. In contrast, the large multicentre ADOPT study found that participants with LADA and type 2 diabetes were equally overweight/obese [39]. In this study, however, GADA was measured in individuals with prevalent diabetes without insulin treatment within the first 3 years of diagnosis. As such, these individuals with LADA were likely to have a more type-2-like phenotype. These findings highlight the heterogeneous nature of LADA and the need for a unified definition.

BMI was positively associated with insulin resistance in LADA, suggesting that this is an underlying pathway. In contrast, there was nothing to suggest that excessive weight would influence autoimmunity per se; there was an inverse association between BMI and GADA level similar to a previous report [25]. Reports of type 1 diabetes in children are in keeping with our data; obesity has been associated with insulin resistance [40], but not with autoimmunity, irrespective of number and type of diabetes antibodies in the study participants [41]. Our findings fit with the accelerator hypothesis [3], which proposes that insulin resistance plays a role in promoting autoimmune diabetes by increasing the insulin demand—this may accelerate disease onset in individuals with an ongoing autoimmune process. In the case of mild autoimmunity, one can hypothesise that factors related to insulin resistance are more important for progression to overt diabetes. This could explain why we found a stronger association between high BMI and less autoimmune LADA and also why the phenotype of the obese individuals with LADA compared with those with normal weight, in line with previous reports [13, 16–18, 25], was more type-2-like, with higher C-peptide levels, better beta cell function and a higher level of insulin resistance. There was a tendency towards a U-shaped relationship between LADA and BMI. If not occurring by chance, it may reflect the weight loss often seen in individuals with type 1 diabetes prior to diagnosis as a consequence of insufficient insulin production.

The association between BMI and LADA was stronger in the prospective data from HUNT, where BMI was assessed several years prior to diagnosis, than in the Swedish case–control data, where BMI was assessed at time of diagnosis. It is possible that the baseline measurements in HUNT reflect a more aetiologically relevant exposure window. Self-reported weight in the case–control study may also have contributed to dilution of associations. On the other hand, the association between type 2 diabetes and BMI was stronger in ESTRID. Another explanation may be that the LADA populations differ in either genetic or unmeasured phenotypical factors.

We confirm the strong association previously reported of overweight and obesity with type 2 diabetes [1]. In addition, we confirm that the combination of overweight and FHD dramatically increases the risk of type 2 diabetes [21] and show, for the first time, that the risk of LADA increases substantially in individuals with FHD and overweight, although the effect is not as pronounced as for type 2 diabetes. Unfortunately, the numbers did not allow us to explore interaction with BMI separately in individuals with a family history of type 1 diabetes vs those with a family history of type 2 diabetes. We have previously shown that LADA is associated with a family history of type 2 diabetes, but even more so with a family history of type 1 diabetes [42], which is in line with genetic studies showing a strong link between LADA and HLA genotypes associated with type 1 diabetes [12]. Together with the findings of present study, this supports the idea that LADA is a hybrid form of diabetes promoted by genes associated with autoimmunity and lifestyle factors inducing insulin resistance.

### Strengths and limitations

The strengths of this study include the large number of incident cases, detailed information on potential confounders and the use of two well-defined population-based studies. The specificity of the GADA assessment was high, but it is possible that some participants with type 2 diabetes were misclassified as LADA, i.e. were false positives. This may contribute to an association with BMI, especially for LADA^low^. It has also been suggested that individuals with LADA and low GADA are actually false positives [43]. However, we found that these individuals differ from those with type 2 diabetes in several clinical characteristics. Also, previous studies in the HUNT Study indicate a real impact of even low and transient levels of GADA, e.g. individuals with low GADA display lower fasting C-peptide levels than individuals with type 2 diabetes [44]. Still, the importance of GADA positivity for disease progression in very obese individuals with low GADA levels is unclear. The sensitivity of the method and the use of only one autoantibody imply that some individuals with LADA were classified as GADA negative, i.e. as having type 2 diabetes. Importantly, GADA is by far the most common autoantibody in LADA, present in ~90% of all individuals [45]. In the HUNT Study, some individuals had GADA measured several years after diagnosis. Because GADA can disappear after prolonged disease duration [44], it is possible that some individuals with LADA therefore appeared GADA negative and were classified as having type 2 diabetes. Notably, GADA tends to be more stable in LADA than in type 1 diabetes [12]. Although there is no unified definition of LADA, the present report is consistent with currently used criteria [12], with the exception of C-peptide which was used in ESTRID as an indicator of remaining insulin production and can be considered a more objective measure compared with the frequently used insulin criterion, i.e. lack of insulin treatment 6–12 months after diagnosis [12]. Estimation of PAR is based on the assumptions of causality and the absence of measurement errors and bias and should hence be interpreted with caution. As for generalisability, it should be noted that PAR is based on the estimated effect size as well as prevalence of overweight in the population and is, as such, population specific. The study is based on populations in Scandinavia, where the incidence of autoimmune diabetes is high, and the results may be less generalisable to areas with lower incidence. Last, assessment on insulin resistance was based on HOMA and even though HOMA has been validated against the hyperinsulinaemic–euglycemic clamp with good correlation [46], it is still a crude method.

In conclusion, under the assumption of causality, excessive weight is a strong contributor to development of LADA and maintaining a healthy weight should be a priority, especially in the presence of FHD or autoimmunity. As expected, obese individuals with LADA had a more type-2-like phenotype, but overweight/obesity was also associated with more autoimmune LADA. These findings support the hypothesis that even in the presence of autoimmunity, factors linked to insulin resistance such as excessive weight could promote the onset of diabetes.

## Electronic supplementary material

ESM Tables (PDF 150 kb)

## Data Availability

The datasets analysed during the current study are available from the corresponding author on reasonable request (ESTRID) and with permission of the HUNT Study by applying to the HUNT Study data access committee.

## Abbreviations

ANDIS: All New Diabetics In Scania
ANDiU: All New Diabetics in Uppsala County
ESTRID: Epidemiological Study of Risk Factors for LADA and Type 2 diabetes
FHD: Family history of diabetes
GADA: GAD antibody
HUNT Study: Nord-Trøndelag Health Study
LADA: Latent autoimmune diabetes in adults
LADA^high^: LADA group with high median GADA
LADA^low^: LADA group with low median GADA
PAR: Population-attributable risk
WHtR: Waist-to-height ratio
