# Supplementary material for: Overweight, obesity and the risk of LADA: results from a Swedish case–control study and the Norwegian HUNT Study
Source: Diabetologia. 2018 Mar 27;61(6):1333–43. doi: 10.1007/s00125-018-4596-0 (PMC6448998; doi:10.1007/s00125-018-4596-0)
Supplement: Supplementary file 1 — (PDF 150 kb) [file 125_2018_4596_MOESM1_ESM.pdf]

ESM Table 1 Characteristics of LADA patients by median GADA-level (196.0 U/ml).
Results from ESTRID, 2010-2016

Characteristics	$LADA^{high}$	$LADA^{low}$	*p*
Number of individuals	197	197	
Men, %	48.7	57.9	*0.0691*
Age, years, mean (SD)	57.4 (12.6)	60.3 (11.9)	*0.0220*
BMI, kg/m^2, mean (SD)	27.3 (5.0)	29.0 (5.4)	*0.0017*
Any first-degree FHD, %	44.2	44.7	*0.9192*
FHD-T2D, %	34.5	38.6	*0.4026*
FHD-T1D, %	12.7	7.6	*0.0953*
With insulin treatment, %	51.0	32.8	*0.0003*
C-peptide, nmol/l, median (IQR)	0.56 (0.50)	0.90 (0.82)	*<0.0001*
HOMA-B, median (IQR)	23.2 (44.4)	46.8 (53.3)	*<0.0001*
HOMA-IR, median (IQR)	2.30 (2.4)	3.10 (2.5)	*0.0017*
HOMA-S, median (IQR)	44.0 (36.5)	32.1 (24.6)	*0.0017*
High-risk HLA, %[a]	70.1	53.8	*0.0012*
Low-risk HLA, %[b]	12.5	30.7	*<0.0001*

[a] Genetic information was available for 93.9 %

T1D, type 1 diabetes; T2D, type 2 diabetes

ESM Table 2 Overweight/obesity and LADA, by sex. Results from ESTRID 2010–2016 and HUNT 1984–2008

	All			Men			Women		
Variable	No. control participants/ person-years	No. Cases	OR/HR[a] (95% CI)	No. control participants/ person-years	No. Cases	OR/HR[a] (95% CI)	No. control participants/ person-years	No. Cases	OR/HR[a] (95% CI)
ESTRID									
BMI (kg/m^2)									
<25	777	125	1	292	63	1	485	62	1
25-29.9	671	165	1.38 (1.06, 1.80)	400	96	1.01 (0.70, 1.46)	271	69	1.83 (1.24, 2.71)
≥30	256	135	2.93 (2.17, 3.97)	128	72	2.45 (1.60, 3.76)	128	63	3.32 (2.13, 5.16)
Per 1 kg/m^2 increase	1704	425	1.10 (1.07, 1.13)	820	231	1.08 (1.04, 1.12)	884	194	1.12 (1.08, 1.16)
Per 1 kg/m^2 increase since age 20	1412	339	1.10 (1.07, 1.14)	678	187	1.08 (1.03, 1.12)	734	152	1.11 (1.06, 1.17)
HUNT									
BMI (kg/m^2)									
<25	494,231	26	1	207,861	12	1	286,370	14	1
25-29.9	401,641	64	2.16 (1.36, 3.43)	221,579	38	2.29 (1.19, 4.41)	180,062	26	2.07 (1.07, 4.02)
≥30	117,085	57	6.07 (3.76, 9.78)	44,488	21	5.92 (2.88, 12.17)	72,597	36	6.37 (3.34, 12.13)
Per 1 kg/m^2 increase	1,012,957	147	1.16 (1.13, 1.20)	473,928	71	1.19 (1.12, 1.25)	539,029	76	1.15 (1.11, 1.20)
Per 1 kg/m^2 increase over time[b]	359,732	48	1.18 (1.03, 1.34)	166,660	24	1.20 (0.96, 1.50)	193,072	24	1.16 (0.99, 1.36)
WHR[c]									
<0.85 (W), <0.90 (M)	329,347	27	1	117,406	10	1	211,941	17	1
≥0.85 (W), ≥0.90 (M)	134,323	24	1.89 (1.03, 3.46)	96,438	16	1.67 (0.74, 3.77)	37,885	8	2.19 (0.93, 5.15)
WHtR[c]									
<0.50	229,054	11	1	80,924	4	1	148,130	7	1
≥0.50	234,035	40	3.14 (1.56, 6.30)	132,737	22	3.01 (1.01, 8.94)	101,298	18	3.22 (1.31, 7.92)

[a] ORs (ESTRID) and HRs (HUNT) adjusted for age, sex, FHD, smoking and physical activity

[b] Change in BMI from HUNT1 (1984-1986) until baseline in HUNT2 (1995-1997)

[c] Information only available from baseline at HUNT2 (1995-1997)

M, men; No., number; W, women

ESM Table 3 Overweight/obesity and Type 2 diabetes, by sex. Results from ESTRID 2010–2016 and HUNT 1984–2008

	All			Men			Women		
Variable	No. control participants/ person-years	No. Cases	OR/HR[a] (95% CI)	No. control participants/ person-years	No. Cases	OR/HR[a] (95% CI)	No. control participants/ person-years	No. Cases	OR/HR[a] (95% CI)
ESTRID									
BMI (kg/m^2)									
<25	777	101	1	292	60	1	485	41	1
25-29.9	671	590	5.14 (3.99, 6.61)	400	389	4.12 (2.92, 5.81)	271	201	7.13 (4.85, 10.48)
≥30	256	729	18.88 (14.29, 24.94)	128	414	14.97 (10.12, 22.14)	128	315	25.29 (16.73, 38.21)
Per 1 kg/m^2 increase	1704	1420	1.29 (1.26, 1.32)	820	863	1.27 (1.23, 1.31)	884	557	1.30 (1.26, 1.35)
Per 1 kg/m^2 increase since age 20	1412	1103	1.27 (1.23, 1.30)	678	670	1.22 (1.17, 1.26)	734	433	1.32 (1.27, 1.38)
HUNT									
BMI (kg/m^2)									
<25	494,231	238	1	207,861	129	1	286,370	109	1
25-29.9	401,641	915	3.30 (2.86, 3.81)	221,579	553	2.97 (2.45, 3.61)	180,062	362	3.70 (2.98, 4.60)
≥30	117,085	849	9.83 (8.49, 11.38)	44,488	373	8.89 (7.26, 10.89)	72,597	476	10.77 (8.69, 13.35)
Per 1 kg/m^2 increase	1,012,957	2002	1.19 (1.18, 1.20)	473,928	1055	1.22 (1.20, 1.24)	539,029	947	1.17 (1.16, 1.18)
Per 1 kg/m^2 increase over time[b]	359,732	943	1.22 (1.18, 1.25)	166,660	493	1.29 (1.23, 1.35)	193,072	450	1.18 (1.14, 1.22)
WHR[c]									
<0.85 (W), <0.90 (M)	329,347	388	1	117,406	126	1	211,941	262	1
≥0.85 (W), ≥0.90 (M)	134,323	650	3.57 (3.11, 4.09)	96,438	418	3.08 (2.52, 3.78)	37,885	232	3.95 (3.30, 4.74)
WHtR[c]									
<0.50	229,054	134	1	80,924	50	1	148,130	84	1
≥0.50	234,035	903	5.08 (4.21, 6.12)	132,737	494	4.31 (3.21, 5.79)	101,298	409	5.53 (4.34, 7.03)

[a] ORs (ESTRID) and HRs (HUNT) adjusted for age, sex, FHD, smoking and physical activity
[b] Change in BMI from HUNT1 (1984-1986) until baseline in HUNT2 (1995-1997)
[c] Information only available from baseline at HUNT2 (1995-1997)
M, men; No., number; W, women

ESM Table 4 Characteristics of LADA patients by BMI category. Results from ESTRID, 2010-2016

Characteristics	BMI<25	BMI 25-29.9	BMI ≥30	*p*[a]
Number of individuals	125	165	135	
Women, %	50.4	58.2	53.3	*0.6362*
Age, years, mean (SD)	58.2 (13.4)	60.4 (12.0)	57.9 (11.4)	*0.8182*
Any first-degree FHD, %	46.4	41.8	48.2	*0.7779*
FHD-T2D, %	35.2	34.6	40.7	*0.3580*
FHD-T1D, %	16.8	9.1	8.9	*0.0555*
With insulin treatment, %	65.3	38.0	22.6	*<0.0001*
C-peptide, nmol/l, median (IQR)[b]	0.37 (0.40)	0.72 (0.54)	1.10 (0.76)	*<0.0001*
GADA, U/ml, median (IQR)	250 (171)	195 (217)	51 (232)	*<0.0001*
HOMA-IR, median (IQR)[b]	1.90 (1.20)	2.55 (2.80)	3.70 (2.20)	*<0.0001*
HOMA-β, median (IQR)[b]	18.7 (35.6)	33.0 (48.9)	56.1 (61.5)	*<0.0001*
HOMA-S, median (IQR)[b]	52.7 (30.8)	39.4 (32.5)	27.3 (18.8)	*<0.0001*
High-risk HLA, %[c]	64.1	62.5	57.5	*0.2980*
Low-risk HLA, %[c]	12.8	22.4	30.0	*0.0013*

[a] *p* for difference between BMI<25 and BMI ≥30

[b] Clinical information was available for 92.7 %

[c] Genetic information was available for 91.5 %

T1D, type 1 diabetes; T2D, type 2 diabetes

ESM Table 5 Characteristics of LADA patients by BMI category. Results from HUNT, 1984-2008

Characteristics	BMI<25	BMI 25-29.9	BMI ≥30	*p*[a]
Number of individuals	26	64	57	
Women, %	53.9	40.6	63.2	*0.4214*
Age, years, mean (SD)[b]	49.4 (12.2)	54.9 (10.7)	56.2 (10.8)	*0.0130*
Age at onset, years, mean (SD)	56.9 (12.9)	59.9 (10.4)	61.4 (11.0)	*0.1091*
WHR, mean (SD)[c]	0.82 (0.10)	0.88 (0.05)	0.90 (0.06)	*0.0168*
Any first-degree FHD, %	38.5	54.7	45.6	*0.5420*
With insulin treatment, %	33.3	17.2	8.9	*0.0180*
C-peptide, nmol/l, median (IQR)[d]	0.16 (0.45)	0.56 (0.66)	0.89 (0.95)	*0.0003*
GADA, U/ml, median (IQR)	215 (413.9)	120.9 (451.5)	80.6 (559.0)	*0.2082*
HOMA-IR, median (IQR)[d]	1.50 (1.90)	1.80 (1.25)	2.50 (2.00)	*0.1820*
HOMA-β, median (IQR)[d]	36.8 (43.2)	55.8 (31.2)	76.3 (80.4)	*0.2118*
HOMA-S, median (IQR)[d]	65.6 (65.5)	55.7 (46.9)	40.7 (36.1)	*0.1644*

[a] *p* for difference between BMI<25 and BMI ≥30
[b]Age at baseline
[c] Information only available from baseline at HUNT2 (1995-1997)
[d] Clinical information was available for 80.3 %
T1D, type 1 diabetes; T2D, type 2 diabetes
